# “Reality” of near-death-experience memories: evidence from a psychodynamic and electrophysiological integrated study

**DOI:** 10.3389/fnhum.2014.00429

**Published:** 2014-06-19

**Authors:** Arianna Palmieri, Vincenzo Calvo, Johann R. Kleinbub, Federica Meconi, Matteo Marangoni, Paolo Barilaro, Alice Broggio, Marco Sambin, Paola Sessa

**Affiliations:** ^1^Department of Philosophy, Sociology, Pedagogy and Applied Psychology (FISPPA), University of PadovaPadova, Italy; ^2^Department of Developmental Psychology and Socialization (DPSS), University of PadovaPadova, Italy; ^3^Centre for Cognitive Neuroscience, University of PadovaPadova, Italy

**Keywords:** NDE, near-death-experience, hypnosis, memory, EEG, integrative effort, clinical psychology, psychophysiology

## Abstract

The nature of near-death-experiences (NDEs) is largely unknown but recent evidence suggests the intriguing possibility that NDEs may refer to actually “perceived,” and stored, experiences (although not necessarily in relation to the external physical world). We adopted an integrated approach involving a hypnosis-based clinical protocol to improve recall and decrease memory inaccuracy together with electroencephalography (EEG) recording in order to investigate the characteristics of NDE memories and their neural markers compared to memories of both real and imagined events. We included 10 participants with NDEs, defined by the Greyson NDE scale, and 10 control subjects without NDE. Memories were assessed using the Memory Characteristics Questionnaire. Our hypnosis-based protocol increased the amount of details in the recall of all kind of memories considered (NDE, real, and imagined events). Findings showed that NDE memories were similar to real memories in terms of detail richness, self-referential, and emotional information. Moreover, NDE memories were significantly different from memories of imagined events. The pattern of EEG results indicated that real memory recall was positively associated with two memory-related frequency bands, i.e., high alpha and gamma. NDE memories were linked with theta band, a well-known marker of episodic memory. The recall of NDE memories was also related to delta band, which indexes processes such as the recollection of the past, as well as trance states, hallucinations, and other related portals to transpersonal experience. It is notable that the EEG pattern of correlations for NDE memory recall differed from the pattern for memories of imagined events. In conclusion, our findings suggest that, at a phenomenological level, NDE memories cannot be considered equivalent to imagined memories, and at a neural level, NDE memories are stored as episodic memories of events experienced in a peculiar state of consciousness.

## Introduction

Near-death-experience (NDE) is classically described as an intense psychological experience of debated nature, characterized by an atypical state of consciousness occurring during an episode of apparent unconsciousness and usually in life-threatening conditions (Moody, 1975; Greyson and Stevenson, 1980). The interest in this phenomenon is well-founded when considering its incidence, ranging from 10% (Greyson, 2003) to about 35% (Zingrone and Alvarado, 2009) in individuals who have been close to death—and its across-the-board nature such that NDE reports in different cultures are substantially similar independently of sociological, demographic, and psychological variables (Kellehear, 2009). It is notable that there is evidence of NDEs in very young children as well (Sutherland, 2009).

In a referential review, Agrillo (2011) outlined the most recurring features reported in the literature that characterize NDEs, namely (a) awareness of being dead; (b) increase of mood in terms of euphoria, happiness, and well-being; (c) out-of-body experience; (d) entering a tunnel-like structure; (e) perception of a light; (f) perception of heavenly or hellish landscape; (g) encounter with deceased relatives, religious figures, or beings of light; (h) experience of a life review; (i) different temporal perception; and (j) perception of sounds or music. These features are included in the NDE scale (Greyson, 1983) currently considered the gold standard to identify veridical NDEs.

An additional facet of this phenomenon, that needs to be mentioned, is that even if a NDE usually occurs in circumstances of closeness to death, the literature describes many reports of individuals that have had a NDE without being physically in danger. A NDE can also be experienced during depression, isolation, meditation (Owens et al., 1990; van Lommel, 2010, 2011), psychological critical life events (Facco and Agrillo, 2012), or it may occur in healthy individuals present during a close relative's death (Moody and Perry, 2010).

Moody's first modern report about NDE, which contains 150 narratives, is entitled “Life after life” (Moody, 1975). This title is illustrative of the challenge, from both scientific and philosophical points of view, represented by NDE as one of the most fascinating issues in cognitive neuroscience research (Agrillo, 2011). In this context, two theoretical frameworks are aimed at interpreting this phenomenon: the so-called “biological/psychological” and the “survivalist” hypotheses (Braithwaite, 2008). The former theory suggests that NDEs are a consequence of brain functional changes that usually, but not always, occurs during the dying process and/or a psychological response to the perceived threat of death, such as cerebral hypoxia (Blackmore and Troscianko, 1988), cerebral anoxia (Lempert et al., 1994), hypercarbia (Klemenc-Ketis et al., 2010), serotonin (Morse et al., 1989) or noradrenaline (Mobbs and Watt, 2011) release alteration, massive liberation of glutamate (Jansen, 1989) or endorphins (Sotelo et al., 1995). In this vein, a referential article by Blanke et al. (2004) reported, for instance, that a typical feature of NDE, i.e., the out-of-body experience, is underpinned by a neural dysfunction, and can occur in neurological patients where the occurrence of a disintegration between personal (vestibular) space and extrapersonal (visual) space is conceivable. The latter theory advances suppositions toward a separation between mind and body, postulating a persistence of some sort of “soul” after the body's death. Among the survivalist interpretations, the theory of continuity proposes that memories, self-identity, and cognition, with emotions, continue to function independently from the unconscious body (van Lommel, 2011; Bókkon et al., 2013).

The numerous reports of NDEs in the absence of life-threatening conditions encourage an extensive, large-scale effort in NDE rendering, that goes beyond the reductionist interpretation that consider NDEs as consequences of dying brain biological mechanisms. One of the most critical aspects that could lead to a further step in understanding such a complex phenomenon is related to NDE's mnesic encoding, storage, and recall. In his review, van Lommel (2011) highlighted some core issues arising from NDE, including “why is the experience of the self during NDE so real? How is it possible to experience enhanced consciousness with the possibility of veridical perception independently of the lifeless body?” (p. 20). NDE memories are characterized by a sense of “phenomenological certainty” of the experience, typical of the perception during daily life (Dell'Olio, 2010). In this vein, Potts (2002) reported that NDEs have been described by individuals as perceived as “real” or even “realer than real.”

In a recent study, Thonnard et al. (2013) examined this aspect of NDEs in depth. The authors specifically investigated NDE memories with the Memory Characteristics Questionnaire (MCQ; Johnson et al., 1988), built to evaluate peculiar characteristics of real and imagined event memories (Johnson et al., 1988). They compared memories of real and imagined past events in three groups of individuals that survived from the coma, one of which was characterized as having had NDEs and a control group. In summary, Thonnard et al. (2013) reported that NDE memories have more features than any kind of memory of real and imagined events, or memories related to a period of totally unconscious state such as coma. According to their interpretation, these findings demonstrate that NDEs cannot be considered as imagined events at all. Moreover, the authors highlighted that NDE memories seem to be *unique* and *unrivaled*.

Given the theoretical relevance of these premises, we were inspired by Thonnard et al. (2013)'s study. Our purpose was to investigate the features of NDE memories both at psychological and electrophysiological levels, in a group of NDE experiencers (NDErs), compared to a matched control group of individuals who never experienced a NDE. In contrast to the study of Thonnard et al. (2013), to achieve our aims we used a hypnotic-based protocol devised to facilitate the richness and thoroughness of the recalls. Such a method, rooted in psychodynamic tradition, is considered an excellent aid to facilitate and to focus on the recall of any kind of memory, both real and imagined (Erdelyi, 1994; Orne et al., 1996; Scoboria et al., 2002; Wagstaff et al., 2004). Particularly worthwhile for our purposes, hypnosis has already been successfully used in clinical practice, as reported in the literature, to evoke memories of NDEs that have happened previously. It was reported that some individuals were able to remember their NDEs only under hypnosis (Schroeter-Kunhardt, 1993; Facco, 2012).

From a psychological perspective, our first objective was to further characterize the NDE memories at the phenomenological level. We analyzed and compared NDE memories with memories of real and imagined events, in a group of NDErs and in a control group. Memory characteristics were assessed using MCQ before (to obtain a common baseline) and after the hypnotic procedure. In accordance with Thonnard et al. (2013), we expected that NDE memories would show similar features to those of real events, and different to those of imagined events.

From an electrophysiological perspective, our hypnosis-based recall approach was implemented by recording the electroencephalographic signals of participants. Electroencephalography (EEG) was chosen as an eligible means to measure neural activity associated with the nature and/or distinctiveness of the NDE memories, when compared to both real and imagined memories. Among the number of advantages that it brings, EEG offered the possibility to explore the neural correlates in a non-invasive way. It permitted a setting where the hypnotist could remain beside the participant during the administration of the hypnotic protocol.

We were particularly interested in examining whether the subjectively perceived peculiarity/vividness of NDE memories had a neural counterpart. Moreover, we were interested in uncovering a specific marker of NDE memories, in line with NDErs' peculiar phenomenological reports. We expected to observe more commonalities between real and NDE memories compared to memories of imagined events. Of particular interest for the present investigation, were those EEG frequency bands labeled theta, alpha (principally the faster frequencies within this band, i.e., high alpha or upper alpha), and gamma, because several EEG studies linked them, among other cognitive processes, to mnesic operations (Bastiaansen and Hagoort, 2003; Jensen et al., 2007; Klimesch, 2012). Cortical theta band oscillations, in the range of 4–7 Hz, observed at frontal, temporal and posterior regions of the scalp have been linked to retrieval in memory paradigms (Burgess and Gruzelier, 2000; Klimesch et al., 2001). Alpha band oscillations, in the range of 7.5–13 Hz, represent the dominant frequency at rest and they mostly originate from the occipital lobe. Klimesch (2012) argued that high alpha band desynchronization primarily reflects controlled access to/retrieval from the knowledge system, including not only long-term memory but also procedural and implicit-perceptual knowledge. Within this theoretical framework, it was suggested that the retrieval of semantically well-integrated information elicits more cortical excitation (i.e., alpha-band desynchronization or decrement of alpha power) than less integrated information. In conclusion, gamma band oscillations (above 30 Hz) also seem related to memory processes. In paradigms exploring long-term memory, it has been shown that gamma activity at the encoding predicts successful memory performance (Sederberg et al., 2003) and at retrieval, gamma activity was stronger for familiar words correctly recognized as having been previously presented than for new words that were correctly rejected as not having been previously presented (Osipova et al., 2006). It is notably that Sederberg et al. (2003) proposed that gamma activity may represent a marker of true memories; thus, during memory recall enhanced “gamma activity” may reflect the reactivation of “synaptically encoded representations” (Jensen et al., 2007) or, in other terms, the reactivation of the neural circuit originally recruited during encoding (Slotnick and Schacter, 2004).

Studies investigating EEG markers of hypnosis have produced mixed results so far (Oakley, 2008; Cardeña et al., 2013), indicating high alpha (MacLeod-Morgan and Lack, 1982), theta (Graffin et al., 1995), and gamma (Cardeña et al., 2013) as potential indices of hypnosis or hypnotic susceptibility. Because our purpose was not to focus on exploring the nature of hypnosis in itself and its neural correlates and because all the critical comparisons we were interested in were between types of memory (retrieved under hypnosis), we decided to focus our analyses on EEG recorded during hypnosis.

In the light of such premises, we expected that silent free recall of (at least) real memories would have been associated with theta, high alpha and/or gamma band power.

We also hypothesized a relationship between the amounts of additional memory details reported after hypnosis and the power of these frequencies. As discussed above, because high alpha band desyncronization is linked to memory processing, we expected to observe a negative correlation between high alpha band power and the difference score in the amount of detail of the real memories after hypnosis. In contrast, a positive correlation was predicted between theta and gamma band power and the difference score for these memories, as suggested by existing evidence. The most intriguing question, however, was whether a similar relation would have been observed also for NDE memories in the experimental group as well. If these memories have characteristics similar to those of real memories and involve the same storage systems, an analogous relationship between theta, high alpha, and/or gamma power and the difference score of NDE memories after hypnosis should be observed. The most appealing issue here was to uncover whether the subjectively perceived *uniqueness* of NDE memories (Thonnard et al., 2013) is associated with qualitatively comparable but eventually enhanced neural processing or rather they are qualitatively different from real memories, and more similar to imagined memories. To better explore this matter we extended our analyses to the other known EEG band frequencies.

According to our knowledge, neural activation in NDErs has been described only in two previous studies, one performed during sleep state (Britton and Bootzin, 2004) and the other in a meditative state/control condition (Beauregard et al., 2009). Therefore, this is the first systematic study that investigates neural activation during NDE recall and, more in general, the first that integrates psychodynamic and electrophysiological techniques.

## Materials and methods

### Participants

Ten individuals who had self-reported NDEs participated in the research. These NDErs were reached by using the referral sampling method, often employed in hidden populations which are difficult for researchers to access (Schroeter-Kunhardt, 1993). They were recruited through a national association composed of individuals who had had NDEs or were just interested in the NDE topic. An advertisement of our research purposes addressed to those who had experienced a NDE was made available by means of a social network, where all the association members were included. Ten of them were reached by phone in order to participate in the study. They were seven females and three males and their mean age was 49.0 years (*SD* = 6.8).

Their NDE was experienced in four of them by a traumatic injury followed by loss of consciousness; three of them reached NDE during a coma state caused by severe medical condition (for example sepsis); two of them had a NDE without any threatening condition (i.e., isolation or existential crisis). NDErs were compared with a 10-subjects control group without NDE.

The control group included 10 volunteer healthy subjects (seven females, mean age = 48.2 years, *SD* = 6.9), matched by gender, age, and educational level with the experimental group.

The first exclusion criterion for all participants was to not report the history of psychiatric, neurological, or substance use disorders and to not take any psychotropic drug at the time of the experimental procedure. The second exclusion criterion was the absence of personality disorders. All participants underwent two in-depth clinical interviews, far-between 1 week. In the first clinical interview, an experienced clinical psychologist administered the Structured Clinical Interview for DSM-IV Axis II Disorder (SCID-II; First et al., 1997) to both groups of participants in order to exclude individuals with personality disorders from the research. In the second clinical interview, two expert psychotherapists investigated through a non-structured interview the presence of personality disorders in order to exclude particularly, subclinical psychotic/schyzotypical traits or dissociative aspects. The only inclusion criterion was related to the experimental group of NDErs. They completed the NDE Scale, a self-rated, 16-item, multiple-choice questionnaire developed to assess these experiences (Greyson, 1983). All of them obtained a score of seven or above, which is generally used as a criterion for considering an experience to be a NDE (Greyson, 1983). The mean NDE score was 16.5 (*SD* = 5.7, range = 7–24). In general, all of the participants met the aforementioned criteria to be considered as eligible to participate in the research. Overall, our study included the participation of 20 subjects, divided into two groups, NDErs and their matched controls.

The study was approved by the Ethics Committee of Padova University (protocol No. 1321). Written informed consent was obtained from all individuals who participated in the study.

### Measures

#### Characteristics of memories

To assess the participants' characteristics of memories, we used the MCQ (Johnson et al., 1988), in a modified version adapted by D'Argembeau and Van der Linden (2008) and used by Thonnard et al. (2013). This MCQ version included 15 items assessing several memory characteristics such as sensory details, memory clarity, self-referential and emotional information, reactivation frequency, and confidence in their own memory (Thonnard et al., 2013). An MCQ total score was derived summing all the 15 items (each on a 1–7 points Likert scale).

#### EEG acquisition and analyses

When populations of neurons simultaneously fire (i.e., synchronization), their rhythmic input is reflected in the extracellular field potential as brain oscillations. Spectral analyses allow decomposing the recorded EEG data by Fast Fourier transform (FFT) into component frequencies. It is largely established that power estimates of each component frequency reflect the number of neural units synchronously active.

EEG activity was continuously recorded under hypnosis during silent free recall of memory conditions. All participants were comfortably seated with their eyes closed. The EEG was recorded from 32 active electrodes placed on an elastic Acti-Cap positioned over the scalp in accordance with an extension of the international 10/20 system and referenced to the left earlobe. Horizontal EOG (HEOG) was bipolarly recorded from electrodes laterally positioned on the outer canthi of both eyes. Vertical EOG (VEOG) was recorded bipolarly from two electrodes, one above (Fp1) and one below the left eye. The impedance was kept less than 10 KΩ. EEG, HEOG, and VEOG activities were amplified (pass band 0.1–100 Hz) and digitized at a sampling rate of 1000 Hz.

EEG was analyzed offline using the Brain Vision Analyzer Software (version 2.0, Brain Products, Munich, Germany). EEG was digitally bandpass filtered (0.5–44 Hz, slope: 24 dB/octave) and re-referenced offline to the average of the left and right earlobes. The filtered and re-referenced EEG was further processed by using an independent component analysis (ICA). We used an ICA-based artifact correction in order to separate and remove VEOG and HEOG artifacts from EEG data by linear decomposition. Components contaminated with eye movements were corrected. EEG was further visually scanned by two independent observers to identify other artifact contamination including muscle artifacts and rejected. Following this procedure data from three participants (two from the experimental group and one from the control group) were excluded from successive analyses because of excessive muscle artifacts. The EEG was segmented into 2000 ms epochs.

For each participant, the artifact-free EEG was then subjected to FFT by averaging over each 2-s epoch using a Hanning window to normalize the spectrum analysis. Spectral power was computed for the following frequency bands: delta (2–3 Hz), theta (3.5–6 Hz), low alpha (7.5–9.45 Hz), mid alpha (9.5–10.45 Hz), high alpha (10.5–12 Hz), beta (14–25 Hz), and gamma (32–44 Hz).

### Procedure

A week before the experimental procedure, all the participants underwent a clinical interview with an experienced clinical psychologist who administered the SCID-II (First et al., 1997) and NDE participants also completed the NDE scale (Greyson, 1983). These measures were collected to exclude the participants with a score on the NDE scale of lower than seven (Greyson, 1983) or those on which, due to physical or mental health issues, hypnosis could have been potentially harmful. Furthermore, following Holden and MacHovec (1993)'s guidelines, the interview was aimed at excluding participants with negative and/or stressful NDE experiences. None of the participants were excluded by these means.

Right before the experimental procedure, we exhaustively described the research methodology, carefully avoiding to explicate the researcher's hypotheses or expectations, and all participants confirmed their agreement. Two trained psychotherapists interviewed participants according to a psychodynamic-oriented approach to confirm their psychological well-being and, mainly, to exclude the presence of psychotic symptoms or high state anxiety levels, according to Holden and MacHovec (1993)'s guidelines. Moreover, the interview was finalized to build a favorable alliance between participants and researchers.

During the experimental procedure we asked participants to recall two distinct memory conditions, in two subsequent sessions, in turn divided in two phases.

The two memories conditions were real events memories and target memories. In detail, in the real events memories condition, participants were asked to recall and narrate the most positive emotionally salient real event memory. In the target memories condition, the NDE group was asked to recall their NDE memory and the control group an imagined event memory (i.e., past dreams, fantasy, or unfulfilled intentions). Participants were invited to match emotional value and personal importance of real memories with target memories.

The first session of the experimental procedure began with a “pre-hypnotic” phase, where subjects were asked to recall the real memory event in a wakeful state, and then to describe it with as much detail as possible. In the second phase, labeled “post-hypnotic” phase, participants were asked to recall the same event after a hypnotic session aimed to enhance richness and thoroughness of memories (Erdelyi, 1994; Scoboria et al., 2002; Wagstaff et al., 2004). At the end of each phase, both “pre-hypnotic” and “post-hypnotic” memories were assessed with MCQ (Jensen et al., 2007; Thonnard et al., 2013).

After the first session, participants were invited to rest for about 15 min and then they were asked to complete an easy visuospatial task, in order to divert their own attention.

Subsequently, in the second session, we repeated the same sequence of “pre-hypnotic” and “post-hypnotic” phases, asking participants to recall target memories.

Real and target memories order was counterbalanced within subjects and between NDE and control groups.

The hypnotic protocol had the same structure in both sessions and was inspired by the protocol used by Palmieri et al. (2012). A trained operator in Eriksonian hypnosis induced a hypnotic state in the participants through a standardized ideomotor general induction, followed by instructions and suggestive symbolic imagery aimed to aid the recall process, including safeguards to avoid a shift from recalling to regressing. When the participants achieved a satisfying hypnotic trance, they were instructed to focus on the memory for 8 min. Further, participants were gently led back to full wakefulness by means of a de-hypnotization process and they were asked to describe the contents of the memory again. All hypnotic procedure was inspired by a psychodynamic, Eriksonian approach to hypnosis. Alongside the whole hypnotic session, encompassing induction and suggestion processes, EEG activity was recorded.

At the end of the second session, the hypnotist operator concluded the protocol according to Holden and MacHovec (1993)'s guidelines for the risk management in hypnotic recall of NDE. This safety procedure included a brief conclusive interview and the administration of the Post-Hypnosis Questionnaire H (Holden and MacHovec, 1993). All the participants in the present study described the hypnotic experience as pleasant or very pleasant (mean pleasantness = 5.9 on a 7-point Likert item) and did not feel any negative, physical, or emotional symptom.

### Statistical analyses

Nonparametric tests were used to compare the amount of detail of memories (MCQ) between experimental and control groups and within different recall conditions. Nonparametric statistical methods were preferred to other parametric statistics because they require few if any assumptions about the shapes of the underlying population distributions and are more robust with small sample size (Siegel and Castellan, 1988; Kitchen, 2009). We used Mann-Whitney U test for between-group comparisons and Wilcoxon Signed Ranks Test for within-group ones. For all analyses, 2-tailed *p*-values of less than 0.05 were considered significant. All analyses were done with SPSS version 18.0.

EEG statistical analyses were conducted on an individual mean value of the spectral power within each frequency band at a subset of pooled electrode sites, resulting in the following regions: frontal (F3/F4), central (C3/C4), parietal (P3/P4), temporal (T7/T8), and occipital (O1/O2). In all multi-factorial analyses (see “EEG spectral power analyses and results”), a Greenhouse-Geisser correction was used where appropriate, i.e., when sphericity assumption was violated (Greenhouse and Geisser, 1959).

## Results

### Characteristics of memories in pre-hypnotic phase

Firstly, we computed the internal consistency of MCQ total score, which resulted in satisfactory scores (Cronbach's alpha of MCQ total score = 0.786).

In order to replicate and extend Thonnard et al. (2013)'s results, we compared MCQ total scores of different memories in the pre-hypnotic phase, considered as a baseline, with the Mann–Whitney U nonparametric between-groups test. Real memories of participants with NDE did not differ from real memories of the control group (*U* = 37.5, *Z* = −0.947, *p* = 0.362), whereas target memories were marginally greater in the experimental group than in the control group (NDEs vs. imagined memories; *U* = 26.0, *Z* = −1.817, *p* = 0.069). In other words, as expected, NDE memories were characterized by a greater amount of detail and richness compared to imagined recalls. In other words, as expected, NDE memories had more characteristics of memories than imagined recalls.

**Figure 1:**
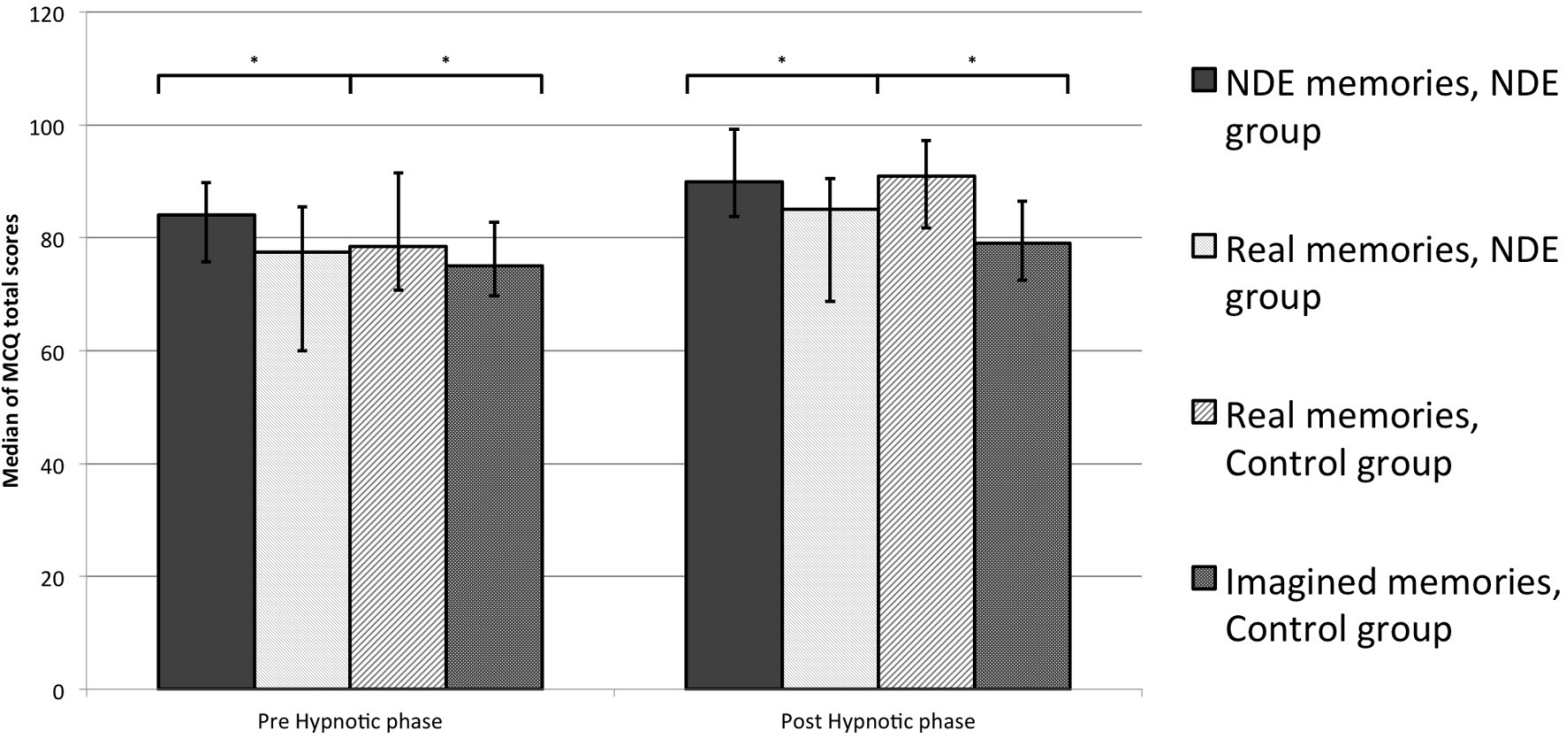
**Memory Characteristics Questionnaire (MCQ) total scores for each assessed memory.** Median and interquartile ranges are presented (^*^p < 0.05).

Within-group comparisons with Wilcoxon Signed Ranks Test showed that in the NDE group there were no significant differences between NDEs and real memories in the pre-hypnotic phase (Wilcoxon *Z* = −1.023, *p* = 0.330). In the control group, real memories were significantly greater than imagined memories (Wilcoxon *Z* = −2.041, *p* = 0.043). Taken together, these results suggest that in the pre-hypnotic phase, the recall of NDE memories and real memories had the same amount of detail and that both were more complex than imagined memories (Figure 1).

### Characteristics of memories in the post-hypnotic phase

The effect of the hypnosis on the characteristics of memories was verified in all experimental conditions (real memories vs. target memories, i.e., NDEs and imagined memories), comparing MCQ total scores collected during the recalls in pre-hypnotic phase with post-hypnotic ones. Hypnosis had a significant effect on memories in all conditions; the amount of detail increased significantly in NDE memories (Wilcoxon *Z* = −2.606, *p* = 0.006), imagined memories (Wilcoxon *Z* = −2.403, *p* = 0.016), and real memories in both groups (Wilcoxon *Z* = −2.809, *p* = 0.002). Moreover, to further understand the effect of hypnosis, we analyzed the difference score (i.e., Δ MCQ) of memory characteristics after hypnosis in all conditions of recall. The difference score was slightly greater in the NDE condition compared to the real condition in the NDE group, but this difference did not reach the statistical significance (median Δ MCQ for NDE memories = 8, median Δ MCQ real memories = 7; Wilcoxon *Z* = −0.767, *p* = 0.443). The difference score was significantly lower in the imagined condition than in the real condition (median Δ MCQ imagined memories = 4; median Δ MCQ real memories = 10; Wilcoxon *Z* = −1.990, *p* = 0.047). Comparison between groups showed no significant differences between NDE and control groups in the difference score for real memories (*U* = 29.0, *Z* = −1.596, *p* = 0.111), and target memories (NDE vs. imagined memories; *U* = 31.0, *Z* = −1.441, *p* = 0.150).

In other words, hypnotic induction proved to be efficacious in all conditions, significantly increasing the complexity and the total amount of detail in all kind of memories. However, the difference score after hypnosis was higher in NDE and real recalls than in imagined ones.

Consistently with these findings, in the post-hypnotic phase we found the same differences among memories that we observed in the pre-hypnotic phase (Figure 1). Real memories did not differ between groups (*U* = 31.0, *Z* = −1.441, *p* = 0.150) whereas there was a significant difference in the target condition. NDE memories significantly resulted in more complexity than imagined memories (*U* = 19.5, *Z* = −2.308, *p* = 0.021). Within-group comparisons showed the same overall picture. NDE memories did not differ from real memories in the NDE group (Wilcoxon *Z* = −1.326, *p* = 0.185); whereas, at the same time in the control group, real memories had greater total scores than imagined ones (Wilcoxon *Z* = −2.448, *p* = 0.014).

### Factorial structure of memory characteristics questionnaire

After having replicated and extended the results of Thonnard et al. (2013), we analyzed the composition of MCQ total score and its structure in depth to better understand neuronal underpinnings of specific kind of memories.

The in-depth analysis of the MCQ items led us to question the adequateness of items 10 and 11 that were on a bipolar scale, and of item 12 and 14 that were not specifically targeted to the recall. Having removed these items, an exploratory factor-analysis led us to hypothesize a four-factor internal structure. We tested such a model through a confirmatory factorial analysis (CFA). The results showed that our model had an overall good fit (χ^2^ = 38.95, *df* = 28, rmsea = 0.07, cfi = 0.95, srmr = 0.06, nnfi = 0.92, bic = 2794.93), and fitted our data better than the original with 1 factor composed of 15 items (χ^2^ = 161.20, *df* = 86, rmsea = 0.11, cfi = 0.77, srmr = 0.10, nnfi = 0.71, bic = 4230.35).

The identified four factors or subscales of the new models were: S1] perceptive and cognitive “resolution” (items 2, 8, and 9); S2] “reliving” (items 3, 13); S3] “veracity” (items 4, 5, and 15); and S4] “spatiotemporal organization” (items 5, 6, and 7). The “resolution” subscale included those items linked to the clarity and richness of the memory; the “reliving” subscale was associated with feelings of re-experiencing the event and the original emotions; the “veracity” subscale was associated with the perception of the memory as veridical and the “spatiotemporal organization” subscale comprised those items associated with the perception of the memory as a well-organized sequence of events.

The scores were averaged to the number of items in each subscale and they were used in the EEG analyses. Items and subscales of modified MCQ are presented in Table 1.

**Table 1:** **Modified version of Memory Characteristics Questionnaire (Thonnard et al., 2013)**.

Item no.	Modified version of Memory Characteristics Questionnaire	Characteristic (Thonnard et al., 2013)	Subscale
1	My memory for this event involves visual details: 1 = none, 7 = a lot	Visual details	NA
2	My memory for this event involves other sensory details (sounds, smells, and/or tastes): 1 = none, 7 = a lot	Other sensory details	S1] perceptive and cognitive resolution
3	While remembering the event, I feel as though I am mentally reliving it: 1 = not at all, 7 = completely	Feeling of re-experiencing	S2] reliving
4	I remember the location where the event took place: 1 = not at all clear, 7 = very clearly	Location	S3] veracity
5	I remember the time of the day when the event took place: 1 = not at all clear, 7 = very clearly	Time	S3] veracity S4] spatiotemporal organization
6	While remembering the event, it comes to me as a coherent story and not as an isolated scene: 1 = not at all, 7 = completely	Coherence	S4] spatiotemporal organization
7	I remember what I did during this event: 1 = not at all, 7 = very clearly	One's own actions	S4] spatiotemporal organization
8	I remember what I said during this event: 1 = not at all, 7 = very clearly	One's own words	S1] perceptive and cognitive resolution
9	I remember what I thought during this event: 1 = not at all, 7 = very clearly	One's own thoughts	S1] perceptive and cognitive resolution
10	Previous studies have shown that people can report that they can visualize different memories from different points of view. Using the bellow mentioned categories, from which point of view do you see yourself? (A) In your memory, you imagine the scene as an observer could see it. As an observer, you can see yourself and other aspects of the situation. (B) In your memory, you imagined the scene from your own point of view (through you own eyes). You are an actor. (C) Any of the above mentioned perspectives described the way you remember the situation. At which point are you observer or actor in the situation: 1 = totally observer; 7 = totally actor	Visual perspective	NA
11	When the event happened, my emotions were: 0 = very negative, 7 = very positive	Valence	NA
12	This event is important to me (it involves an important theme or episode in my life): 1 = not at all important, 7 = very important	Personal importance	NA
13	While remembering the event, I feel the emotions I felt when the event occurred: 1 = not at all, 7 = completely	Feeling emotions	S2] reliving
14	Since it occurred, I have thought or talked about this event: 1 = not at all, 7 = very often	Reactivation frequency	NA
15	I believe the event in my memory really occurred in the way I remember it and that I have not imagined or fabricated anything that did not occur: 1 = 100% imaginary, 7 = 100% real	Real/imagine	S3] veracity

### EEG power spectra analyses and results

The mean of the spectral power within each frequency band was submitted to repeated measure ANOVAs considering region (5 levels: frontal, central, parietal, temporal, and occipital) and condition (NDE/imagined memories vs. real memories) as within-subjects factors, and group (experimental vs. control participants) as between-subjects factor. The individual mean values of the spectral power within each frequency band for each group and in each memory condition were also correlated (i.e., Pearson's *r*) with the difference score in MCQ after hypnosis (i.e., Δ MCQ for NDE memories and Δ MCQ for real memories for the experimental group; and Δ MCQ for imagined memories and Δ MCQ for real memories for the control group). Furthermore, to uncover finer relationships between the spectral power within each frequency band and the difference score after hypnosis, the Δ MCQ for each of the four subscales (“resolution,” “reliving,” “veracity,” and “spatiotemporal organization”) for each group of participants and for each condition, was also correlated with the spectral power within each band.

#### Delta band power (2–3 Hz)

The main effect of region was significant, *F*_(2.396, 12)_ = 23.46, *p* < 0.001, η^2^_*p*_ = 0.610. Bonferroni-corrected multiple comparison post-tests showed that power in the delta band was lower at temporal region than at all other regions (all *p*s < 0.001), and that it was higher at parietal region than occipital region (*p* = 0.014). The interaction condition (i.e., NDE/imagined memories vs. real memories) × group (i.e., experimental vs. control participants) was also significant, *F*_(1, 15)_ = 5.73, *p* = 0.030, η^2^_*p*_ = 0.276. Separate ANOVAs for each group revealed that power in the delta band tended to be different between the imagined memories (mean power 1.733 μV^2^) and the real memories (mean power 1.265 μV^2^) conditions in the control group, *F*_(1, 8)_ = 3.89, *p* = 0.084, η^2^_*p*_ = 0.327. For the experimental group, power in the delta band was positively correlated with the total individual scores of additional NDE memories details recalled following hypnosis (i.e., Δ MCQ for NDE memories) at frontal region, *r* = 0.778, *p* = 0.023, and at temporal region *r* = 0.716, *p* = 0.046. This association was particularly evident with regard to the “resolution” (at frontal region: *r* = 0.785, *p* = 0.010; at temporal region, *r* = 0.712, *p* = 0.024), the “reliving” (frontal region: *r* = 0.712, *p* = 0.018), and “spatiotemporal organization” (at all regions; frontal region: *r* = 0.732, *p* = 0.020; central region: *r* = 0.652, *p* = 0.040; parietal region: *r* = 0.639, *p* = 0.044; temporal region: *r* = 0.842, *p* = 0.004; occipital region: *r* = 0.634, *p* = 0.046) aspects of the NDE memories.

In the control group, a negative correlation was observed between the power in the delta band and the total difference score in imagined memories following hypnosis (i.e., total Δ MCQ for imagined memories) at frontal region, *r* = −0.677, *p* = 0.045, and an analogous trend was observed at central and temporal regions; although, only marginally significant, *r* = −0.624, *p* = 0.072, and *r* = −0.626, *p* = 0.071, respectively.

#### Theta band power (3.5–6 Hz)

The only significant effect was that of region, *F*_(1.992, 12)_ = 15.35, *p* < 0.001, η^2^_*p*_ = 0.506. Bonferroni-corrected multiple comparison post-tests showed that power in the theta band was lower at temporal regions than at all other regions (all *p*s < 0.05). Mean theta band power did not correlate with the total difference score in MCQ score following hypnosis (i.e., Δ MCQ). However, when considering separately MCQ subscales, in the experimental group was observed a positive correlation between the power in the theta band and the “spatiotemporal organization” aspect of the NDE memories (frontal region: *r* = 0.712, *p* = 0.024; temporal region: *r* = 0.614, *p* = 0.053; an analogous trend, although only marginally significant, was also observed at central, parietal, and occipital regions (*r* = 0.539, *p* = 0.084; *r* = 0.547, *p* = 0.080; *r* = 0.513, *p* = 0.097, respectively). An opposite relationship (i.e., negative correlations) was observed in the control group between the power in the theta band and the “resolution” (frontal region: *r* = −0.609, *p* = 0.041; central region: *r* = −0.594, *p* = 0.046; temporal region, *r* = −0.640, *p* = 0.032) and “reliving” (central region: *r* = −0.648, *p* = 0.029; an analogous trend, although only marginally significant, was also observed at parietal, temporal, and occipital region: *r* = −0.568, *p* = 0.055; *r* = −0.541, *p* = 0.066; *r* = −0.551, *p* = 0.062, respectively) of imagined memories.

#### Low alpha band power (7.5–9.45 Hz)

Statistical analyses of mean power in the low alpha band did not show any significant effect (min *p* = 0.196). Mean low alpha band power did not correlate with individual scores of the difference score in MCQ following hypnosis (i.e., Δ MCQ).

#### Mid alpha band power (9.5–10.45 Hz)

Statistical analyses on mean power in the mid alpha band did not show any significant effect (min *p* = 0.491). Mean mid alpha band power did not correlate with individual scores of difference score in MCQ following hypnosis (i.e., Δ MCQ).

#### High alpha band power (10.5–12 Hz)

The main effect of region was significant, *F*_(1.290, 12)_ = 15.67, *p* < 0.001, η^2^_*p*_ = 0.511. Bonferroni-corrected multiple comparison post-tests showed that power in the high alpha band was higher at occipital and parietal regions compared to the other regions and lower at temporal and frontal regions compared to the other regions (max *p* = 0.016). The main effect of group was also significant, *F*_(1, 15)_ = 4.962, *p* = 0.04, η^2^_*p*_ = 0.249, indicating greater power in the high alpha band in the experimental group (mean power 4.51 μV^2^) than in the control group (mean power 1.82 μV^2^). The interaction region × group tended to be significant, *F*_(1.290, 12)_ = 3.72, *p* = 0.059, η^2^_*p*_ = 0.199, indicating that the distribution of the high alpha across the scalp tended to be different in the two groups of participants. Separate ANOVAs for each group revealed that power in the high alpha band tended to be lower at parietal region (mean power 2.54 μV^2^) than at occipital regions (mean power 3.58 μV^2^) in the control group, *p* = 0.075.

For the experimental group, power in the high alpha band was negatively correlated with the total individual scores of the difference score of real memories following hypnosis (i.e., Δ MCQ for real memories) at frontal regions, *r* = −0.713, *p* = 0.047. No individual subscales of the Δ MCQ was particularly linked with alpha power. For the control group, power in the high alpha band was negatively correlated with the “reliving” aspect of the real memories (temporal region: *r* = −0.603, *p* = 0.043; and marginally at frontal region: *r* = −0.534, *p* = 0.069).

#### Beta band power (14–25 Hz)

The main effect of the group was significant *F*_(1, 15)_ = 6.073, *p* = 0.026, η^2^_*p*_ = 0.288, indicating greater power in the beta band in the experimental group (mean power 0.82 μV^2^) than in the control group (mean power 0.37 μV^2^). Mean beta band power did not correlate with the individual scores of the difference score in MCQ following hypnosis (i.e., Δ MCQ).

#### Gamma band power (32–44 Hz)

The main effect of region was significant, *F*_(1.701, 12)_ = 3.67, *p* = 0.026, η^2^_*p*_ = 0.196. Bonferroni-corrected multiple comparison post-tests showed that power in the gamma band was significantly higher at frontal region than at parietal region (*p* = 0.035). For the control group, power in the gamma band was positively correlated with the individual scores of the difference score in MCQ for real memories following hypnosis (i.e., Δ MCQ for real memories) at central regions, *r* = 0.679, *p* = 0.022. A finer-grained analysis considering Δ MCQ subscales revealed a richer pattern of results: first, the correlation observed for the control group was particularly evident for the “resolution” (frontal region: *r* = 0.755, *p* = 0.009; at parietal region, *r* = 0.817, *p* = 0.004; at temporal region, *r* = 0.679, *p* = 0.022; at occipital region, *r* = 0.713, *p* = 0.016), the “veracity” (at central region: *r* = 0.661, *p* = 0.026), and the “spatiotemporal organization” (at central region: *r* = 0.606, *p* = 0.042) aspects of the real memories. Furthermore, for the experimental group this finer analysis revealed a positive correlation between power in the gamma band and the “reliving” (temporal region: *r* = 0.728, *p* = 0.020) aspect of the real memories.

**Figure 2:**
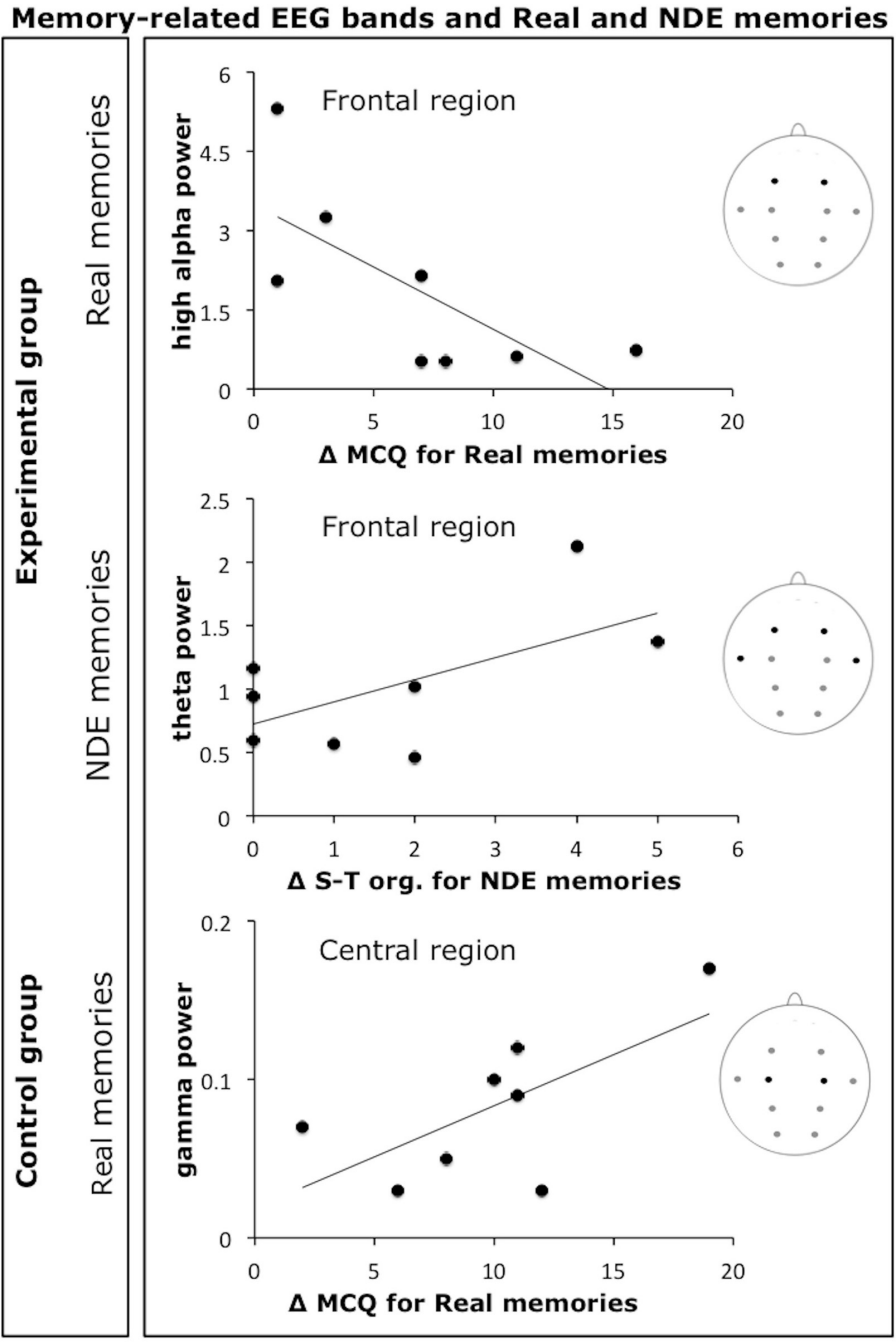
**Scatterplots of a subset of observed correlations between memory-related EEG frequency bands (high alpha, theta, and gamma) and individual Δ MCQ scores or Δ MCQ subscales scores.** Scatterplots show the most relevant correlations for the real memories and the NDE memories conditions in the experimental group and for the real memories condition in the control group (Δ S-T org. = Δ MCQ scores for the spatiotemporal organization subscale).

**Figure 3:**
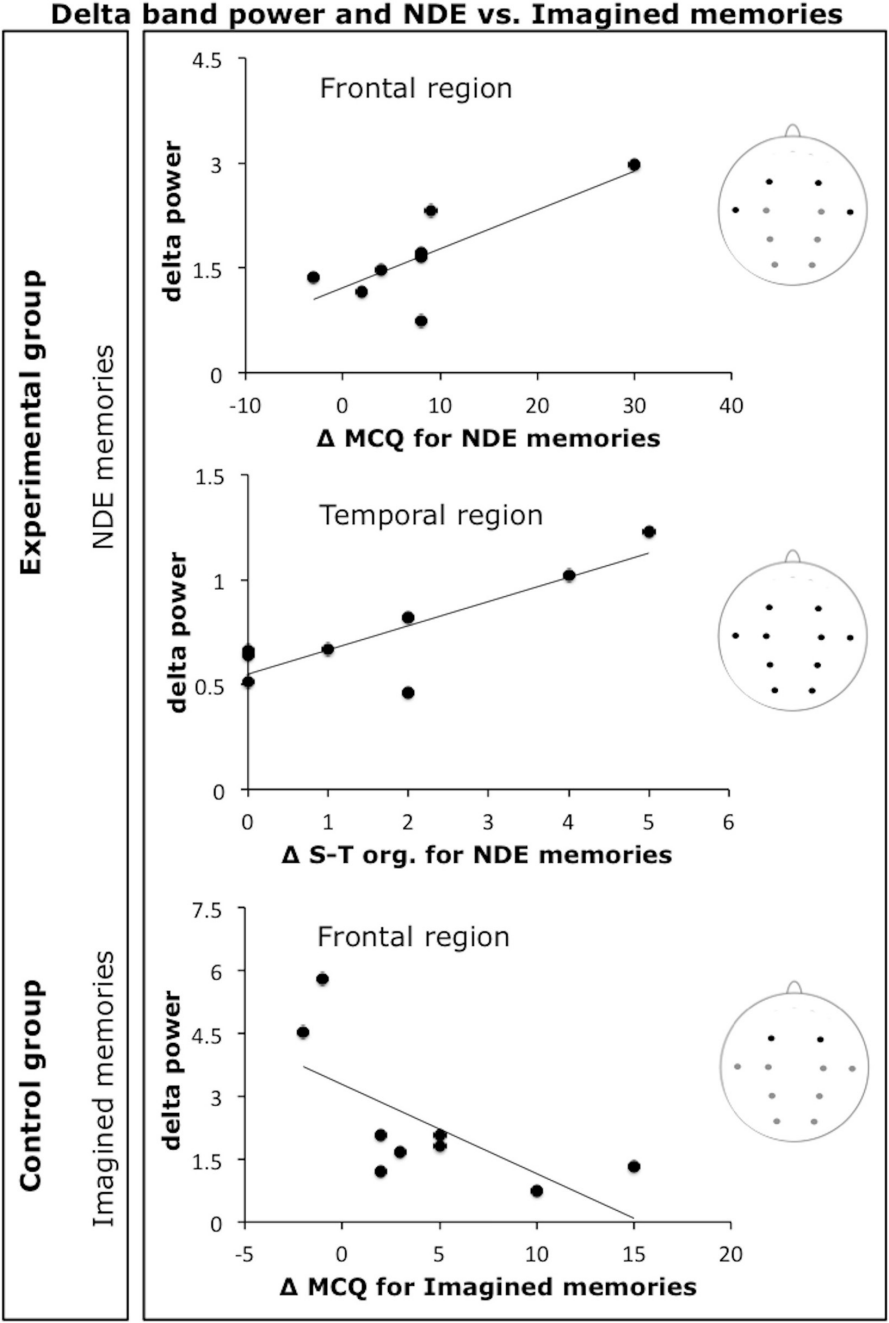
**Scatterplots of a subset of observed correlations between delta band and individual Δ MCQ scores or Δ MCQ subscales scores.** The first two panels show the correlations with the NDE memories condition in the experimental group and the third panel with the imagined Memories condition in the control group (Δ S-T org. = Δ MCQ scores for the spatiotemporal organization subscale).

Figure 2 shows the scatterplots of the most relevant correlations between memory-related EEG frequency bands (high alpha, theta, and gamma) and individual Δ MCQ scores (or subscales scores) for the real memories and the NDE memories conditions in the experimental group and for the real memories condition in the control group. Figure 3 shows the scatterplots of a subset of observed correlations between delta band and individual Δ MCQ scores for the NDE memories condition in the experimental group and for the imagined memory condition in the control group.

Table 2 schematically illustrates the pattern of correlations between each EEG frequency band considered in the present study (delta, theta, low alpha, mid alpha, high alpha, beta, and gamma) and Δ MCQ scores and subscales scores for each group of participants (experimental and control groups) and for each type of memory (Real, NDE, and Imagined).

**Table 2:** **Pattern of correlations between each EEG band and Δ MCQ scores**.

**EEG band**	**Experimental group**		**Control group**	
	**Real memories**	**NDE memories**	**Real memories**	**Imagined memories**
Delta	n.s.	*Positive correlations* with Δ MCQ (at frontal and temporal regions); with Δ MCQ for resolution (at frontal region) and with Δ MCQ for spatiotemporal organization (at all regions). *rs* ranging from 0.634 to 0.842	n.s.	*Negative correlations* with Δ MCQ (at frontal, central, and temporal regions). *rs* ranging from −0.626 to −0.677
Theta	n.s.	*Positive correlations* with Δ MCQ for spatiotemporal organization (at all regions). *rs* ranging from 0.513 to 0.712	n.s.	*Negative correlations* with Δ MCQ for resolution (at frontal, central, and temporal regions) and for with Δ MCQ for reliving (at central, parietal temporal and occipital regions). *rs* ranging from −0.541 to −0.648
Low alpha	n.s.	n.s.	n.s.	n.s.
Mid alpha	n.s.	n.s.	n.s.	n.s.
High alpha	*Negative correlations* with Δ MCQ (at frontal region). *r* = −0.713	n.s.	*Negative correlations* with Δ MCQ for reliving (at frontal and temporal regions). *r* = −0.603 and *r* = −0.534, respectively	n.s.
Beta	n.s.	n.s.	n.s.	n.s.
Gamma	*Positive correlations* with Δ MCQ for reliving (at temporal region). *r* = 0.728	n.s.	*Positive correlations* with Δ MCQ (at central region) and with Δ MCQ for resolution (at frontal and parietal regions). *rs* ranging from 0.606 to 0.817	n.s.

n.s., no significant.

## Discussion

In this study, an integrative effort was employed, from a psychodynamic and electrophysiological perspective, to catch an in-depth insight into NDE recall. It is notable that this experience is typically defined by most NDErs as “realer than real” (Blackmore, 1993; Potts, 2002). To investigate this phenomenon, we compared NDE memories with the memories of real autobiographical events and memories of imagined autobiographical events, in a group of NDErs and in a matched control group. NDE memories were verbally recalled both in pre- and post-hypnotic wakeful state. During hypnosis, the free silent recall was EEG recorded.

From a psychological point of view, our findings were in accordance with Thonnard et al. (2013), which inspired our theoretical premises, and with our first hypothesis. Our expectation was to characterize NDE memories as similar to real memories and as different from imagined memories in terms of mnesic cues that can discern one from the other.

In the baseline condition before hypnotic session, NDE memories revealed more detail than imagined memories, as investigated with MCQ questionnaire (Thonnard et al., 2013). Moreover, according to our hypothesis, in the NDE group there were no significant differences between NDE and real memories; consistently with these findings, in the control group, MCQ total scores of real memories were significantly greater than that of imagined memories. Overall, we found that in the pre-hypnosis phase, the NDE memories and the real memories had the same amount of mnesic characteristics and both were more complex and richer than imagined memories. From a phenomenological perspective, it could be inferred that NDE memories showed high similarities with real memories, and profound dissimilarities from imagined memories. NDErs who participated in our study always considered their NDE memories far superior to their real memories from all points of view, despite the efforts of the experimenter to invite them in finding a comparable event that happened in real life. All participants who lived NDEs described them as the most powerful, intense, vivid, important, and founding experience of their life.

Quotation of a NDE participant to our study: “In 45 years I dreamed a lot of things, but I never had a ‘dream’ like this. (…) When you dream, you know that it is a dream. When I remember my dreams, in the next 5 minutes I'm not sure anymore about their content. This experience (note: NDE) instead, you'll remember it clearly, for years.”

Consistently with the literature (Orne et al., 1996; Scoboria et al., 2002) and with predictions originated by the clinical practice, all kind of memories revealed amelioration thanks to hypnotic sessions. In all the experimental conditions (target memories, i.e., NDE memories and imagined memories, and real memories), in facts, hypnosis had a significant facilitating effect. In other terms, the hypnotic procedure significantly increased the complexity and the total amount of detail of all kind of memories, as measured with MCQ. Consequently, MCQ score obtained in the post-hypnotic phase, maintained the same trend and the same level of significance of the pre-hypnotic phase, revealing that NDE memories features are totally similar, in terms of sensory, clarity, self-referential information emotional, and confidence of real memories, and significantly different from more poorly-detailed imagined events memories.

Findings by Thonnard et al. (2013) also suggested that NDE memories are actually perceived although, not-lived in the external world. The authors concluded their manuscript inviting neural investigation to deepen the roots of this phenomenon perceived as so veracious: albeit, it happened in an unconscious state. In this vein, at the neural level, our second hypothesis was to observe a relationship between recall of real memories and the power of those band oscillations associated with memory functioning recorded during silent free recall under hypnosis, i.e., theta, high alpha, and gamma. The most critical issue was whether a relation with these well-known neural indices of memory would have been observed for NDE memories in the experimental group as well. Given the reported *uniqueness* of NDE memories (Thonnard et al., 2013), we extended our analyses to the other EEG bands, and the Δ MCQ for each of the four subscales derived from the MCQ (i.e., “resolution,” “reliving,” “veracity,” and “spatiotemporal organization”).

In line with our hypotheses, a better recall of real memories (both total additional memory details recalled following hypnosis, i.e., Δ MCQ for each type of memory, for each group, and Δ MCQ for each of the four subscales) was correlated with a pattern of high alpha power decrease/gamma power increase, in both experimental and control groups of participants. These findings correspond well with previous researches showing a link between these frequency bands and long-term memory performance (e.g., Sederberg et al., 2003; Klimesch et al., 2008). It has been proposed that gamma activity may represent a marker of true memories (Sederberg et al., 2003). This hypothesis views gamma activity as an index of the reactivation of the neural circuits originally recruited during encoding, which usually includes the occipital regions originally engaged in the encoding of visual objects and scenes (Slotnick and Schacter, 2004). This relationship between gamma band and real memories also involved the occipital region. Such a relationship was not observed for NDE memories (possibly suggesting that they were the result of an internally generated experience, e.g., hallucination-like form). In contrast, the increase of theta band power at temporal region positively correlated with the recall of the details of NDE memories associated with the perception of the memory as a well-organized sequence of events (i.e., “spatiotemporal organization” subscale). Although evidence is not conclusive at present, it is suggested that cortical theta oscillations are connected to intracranial recorded hippocampal theta oscillations. Because the critical role of hippocampus in memory functioning is well established (Squire, 2009), it was hypothesized that cortical and hippocampal theta oscillations dynamically interact, supporting memory operations (Bastiaansen and Hagoort, 2003).

The most recent theoretical scenario supports the notion that hippocampal theta oscillations are mainly associated with the temporal organization of episodic memories (Buzsáki and Moser, 2013), indirectly proposing that the retrieval of highly organized memories in terms of spatiotemporal sequences may also be linked to an increase in cortical theta power. It is noteworthy that the observed correlation between the power in the theta band and the recall of the details of NDE memories associated with the “spatiotemporal organization” subscale, seems to nicely converge with this hypothesis. Importantly, in the control group, the power in the theta band was negatively correlated with additional details of imagined memories reported following hypnosis, particularly in relation to those memory details associated with the perception of clarity and richness (i.e., “resolution” subscale) and the feeling of re-experiencing the event and the original emotions (i.e., “reliving” subscale).

In conclusion, the above-described pattern of findings indicated that “successful” recall of real memories was related with power in the high alpha/gamma bands. Furthermore, NDE memories were also linked with power in the theta band, a well-known marker of episodic memory. Moreover, the EEG pattern of correlations for NDE memory recall critically differed from that observed for imagined memories. As mentioned before, in order to extensively explore the issue of a neural marker of NDE memories, we decided to extend analyses to the other EEG bands. Of particular relevance are the findings relative to delta band oscillations. Power in this band tended to be different between the real memories and the imagined events memories conditions in the control group, but a comparable power was observed for the real memories and the NDE memories conditions in the experimental group. In two recent reviews on delta activity, Knyazev (2012) and Harmony (2013) indicated a variety of conditions and mental functions associated with delta activity, such as those related to evolutionarily old basic processes, motivation, reward, as well as mental concentration. On the basis of extant evidence, one conceivable interpretation of these findings is that the recall of both real and NDE memories were more rewarding than the recall of imagined memories.

Furthermore, power in the delta band was positively (and selectively) correlated with additional details of NDE memories reported following hypnosis, particularly in relation to those memory details associated with the perception of the memory as clear and detailed (i.e., “resolution” subscale), the feeling of re-experiencing the event and the original emotions (i.e., “reliving” subscale), and the perception of the memory as a well-organized sequence of events (i.e., “spatiotemporal organization” subscale). One explanation of this relationship may refer to the hypothesis that delta oscillations are also indicative of inhibition of sensory afferences, a mechanism that will favor internally directed cognition (i.e., “internal mentation”). This internal mentation includes a recollection of the past (Harmony, 2013). In these terms, an increase in delta power may have supported the recollection of NDE memories. Although reasonable, this interpretation does not account for a potentially relevant aspect of this finding, i.e., the selectivity of the correlation between the power in the delta band and additional details of NDE memories.

A different and maybe more appealing perspective may relate to the notion of “state-dependent memory,” also called “state-dependent learning,” based on which the retrieval of a memory is facilitated by the match between the original state of consciousness experienced at the encoding and the current state of consciousness at retrieval (Overton, 1966). To note, the two perspectives (namely, internal mentation and state-dependent memory) are not mutually exclusive; however they may integrate providing a more comprehensive interpretation of this relation between delta activity and NDE memories. Although evidence is scarce in this regard and sometimes anecdotal, one may speculate that NDE memories were better retrieved when delta power was greater because during the original NDE, delta dominated the EEG pattern, or, at least, it was critically associated with the experience itself. Slow-wave activity has been related to certain trance states and other related portals to transpersonal experience (Hartman and Zimberoff, 2002), including the shamanic state of consciousness, out-of-body experiences, NDEs, and lucid dreaming. Furthermore, Strubelt and Maas (2008) asserted that if, during the NDE, neural circuits (including those of the neocortex) usually engaged in memory encoding are functioning, the experience may be encoded and retrieved later.

As a further argumentation in favor of state-dependent-memory hypothesis, the hypnotic state has seldom been used to evoke previously occurring NDEs; some individuals who experienced this phenomenon were able to remember it only in hypnosis (Schroeter-Kunhardt, 1993). Facco (2012) underlined that some common processes could link the hypnotic state with the NDE experience: both of them have been included in the so-called “altered state of consciousness.” Facco (2012) stated that, although NDE and hypnosis are unequivocally two distinct phenomena, some common processes probably link them. For example, experiences similar to those of NDE can be easily generated during hypnosis, such as (a) imagining seeing oneself from the outside, (b) change in time perception, (c) recalling old and non-easily accessible memories, and (d) performing a life review. Holden and MacHovec (1993) quoted a case of a man submitted to hypnosis, who previously had a NDE during an anaphylactic shock. When he recalled it during a hypnosis session, he replicated the whole experience, including negative physical changes, in terms of sudden fall of arterial blood pressure with a great increase of heart rate (up to 190 beats/min), and he re-entered the normal, physiological state after de-hypnotization. Therefore, although it can only be regarded as speculation, our electrophysiological findings fit very well with the hypothesis of the state-dependent memory, such that an EEG pattern similar to that originally present during the NDEs may facilitate a recall of NDE memories.

To summarize the whole pattern of EEG findings, it appears to unveil a peculiar pattern of neural activity associated with the recall of NDE memories linked to slow-wave activity, including both delta and theta oscillations. Theta power represents a well-known marker of memory processing, particularly in relation to episodic memories and their spatiotemporal organization (Buzsáki and Moser, 2013); delta power has also been associated with internal mentation including the recollection of the past (Harmony, 2013). On the other hand, the recall of these NDE memories did not show any relationship with gamma power, which has been designated as a marker of true memories (Sederberg et al., 2003), where “true” indicates that the memory recall reactivates the sensory circuits originally recruited during encoding of objects, scenes, events experienced in the physical world. Whitton et al. (1978) demonstrated that both unmedicated schizophrenics with Schneiderian criteria during hallucinations and healthy control participants during a “creativity” test exhibited an EEG frequency pattern of predominantly delta and theta power. This whole pattern may fit with the proposal of Thonnard et al. (2013) that NDE memories are hallucination-like memories of actually perceived hallucinations. In fact, in the present investigation, NDE recall was related to both delta (recollection of the past but also trance states and hallucinations) and theta power (episodic memory) but not with gamma power (true memories; experienced in the physical world). In synthesis, the EEG findings suggest that NDE memories are episodic memories of events experienced in a peculiar state of consciousness.

To briefly delineate the intriguing theoretical controversy between psychological/biological and survivalist theorists in the matter of NDE memories, it is necessary to begin from the origin of the debate. In general, it is important to take into account that, according to well-established classical findings, memories of human beings originating from real, everyday life, should have more perceptual information (e.g., color and sound), more contextual information, and more details than memories originated from thought (Johnson et al., 1988). Blackmore (1993) proposed a biological/psychological hypothesis that the emerging memories of NDE could be totally or partially imagined, as a result of peculiar neuronal pattern of activation and a reconstructive cognitive process, immediately after the resuscitation, influenced by personal knowledge and expectations. Psychological studies from forensic fields have shown that simple imagining having had a specific experience can create false memories (Loftus, 2001). In other words, it has been proposed that reports of NDEs could be memories of totally or partially imagined events. Survivalist theorists have argued that false memories are hardly conjugated with rich content information that characterized NDE. Moreover, it would not be conceivable that a neural circuit of resource-poor, damaged brain could support false memory (Parnia et al., 2001). Agrillo (2011) argued that, if the brain is too unstable to support such processes, the question would focus on where the NDE memories would be stored. As Braithwaite (2008) outlined, in order to have any experience to be remembered, the memory should in the first place encode and represent the experience.

Finally, our findings seem inconsistent with the idea that such vivid NDE reports can be originated by totally or partially imagined false memory. In contrast, a suggestive interpretation of our EEG results appears to contradict the classic idea (van Lommel et al., 2001) that during a life-threatening event, such as a cardiac arrest, there is no activity of the cortex and the brainstem (implying a transient loss of all functions of the brain). A recent study by Borjigin et al. (2013) was able to reveal brain activity in rats 30 s after a cardiac arrest by using a finer technique, i.e., intracranial EEG recording. This activity was characterized by a transient surge of synchronous gamma oscillations phase-coupled to both slow-wave and alpha oscillations, and the authors suggested that the whole pattern indexed a heightened conscious processing near-death. However, Greyson et al. (2013), in a subsequent commentary, raised some criticisms about the interpretation by Borjigin et al. (2013) of their above mentioned data. Among these criticisms, the fact that the EEG registered in the rats was a tiny fraction of the total neuroelectric power present before the cardiac arrest could be misleading to consider this EEG activity a neural underpinning of conscious processing. According to Greyson et al. (2013), it is impossible to establish what, if anything, the rats were experiencing during the post-arrest period of the surge. In addition, Borjigin et al. (2013) found that the EEG burst did not occur in anesthetized rats, whereas NDEs commonly occur in people who are anesthetized.

Either way, if NDE memory is related to a real event, it should imply that enough neural activation would be available to encode and represent the experience and subsequently to report it. As mentioned above, our results are in line with the hypothesis that the core components of a NDE have a neural counterpart. If we assume that some physiological mechanisms can account for NDEs, then the individual really perceives what was reported later, albeit not necessarily corresponding to occurring events in the external, physical world. In a very speculative perspective, NDE phenomenon could begin some hours, days, even weeks before the effective exitus, in terms of End of Life Dreams and visions (ELDVs). The hypothesis of common neural mechanism between ELDVs and NDEs, where ELDVs phenomenon is a sort of precursor of NDEs, may not be so implausible. Even if scientific literature describes ELDVs and NDEs as two distinct phenomena, a number of common characteristics [i.e., vivid and memorable visions, encounter with deceased loved ones, feeling of joy and serenity, transcendence, spiritual transformation after the experience, Nosek et al. (2014)] make conceivable that they could be two entities of the same continuum.

Agrillo (2011) labels the psychological/biological and survivalist interpretations as “in brain” or “out of brain” theories, respectively, highlighting that survivalist hypothesis do not necessarily exclude the role of biological or psychological components underlying NDE. According to the author, the crucial point is not whether “something” can survive after biological death, because nobody can say anything for sure in this regard, but whether the NDE phenomenon is explicable at least in terms of brain functioning. In this line, our data supporting the idea of a neural counterpart of the phenomenon, are not necessarily in contrast with a more spiritualistic theory. In fact, our findings suggest a neural support that allowed NDE mnesic storage in a brain that was partially functional (e.g., in coma patients, or in cardiac arrest patients), regardless of a flatline EEG, which measures only surface cortical activity, as suggested by some authors (Bardy, 2002; Braithwaite, 2008; Borjigin et al., 2013) or fully functional (e.g., in isolation condition or meditative state: Owens et al., 1990; van Lommel, 2010, 2011) at the moment when NDE happened. In other words, even if the investigation of neural underpinnings in experiencing or recalling NDE could be roughly ascribed to a psychological/biological position, uncovering the neural counterpart of NDE does not exclude *per se* survivalist hypothesis. It is likely that many of the arguments reported to support the former or the latter antipodal positions would be, in fact, not necessarily mutually exclusive, as in the case of our findings.

The main limitation of our research is the relatively small sample size. However, people who experienced NDE classically represent a hard-to-reach population, because of their reluctance in talking about their experience to avoid negative reactions in the others (Schroeter-Kunhardt, 1993; van Lommel, 2011). Further studies including wider samples are warranted.

In conclusion, our integrative-effort study showed that NDE memories are different from imagined autobiographical memories and very similar to memories of real events, in terms of detail richness, self-referential and emotional information. EEG signal recorded during hypnotic sessions, successfully employed to facilitate memory retrieval, revealed a pattern of neural activation during the NDE recall that seemed to have supported the retrieval of additional details. Such neural signature was characterized by slow-wave activity, including both theta (i.e., a marker of memory processing, particularly related to episodic memories with spatiotemporal organization) and delta (which has also been associated with internal mentation and meditative state) oscillations.

Several possible explicative hypotheses, expounded in this section, could arise from our observations, such as the role of dependent-state-memory in NDE recall during hypnosis. Moreover, our impression is that our results could lead to a little, further step in the direction of an understanding of NDE memories; although, many questions remain unanswered and further research is needed to shed light to this intriguing phenomenon of consciousness.

## Author contributions

Design and conceptualization of the study: Arianna Palmieri, Vincenzo Calvo, Johann R. Kleinbub, Paola Sessa. Acquisition of data: Arianna Palmieri, Vincenzo Calvo, Johann R. Kleinbub, Federica Meconi, Matteo Marangoni, Paolo Barilaro, Alice Broggio, Marco Sambin, Paola Sessa. Statistical analysis: Vincenzo Calvo, Johann R. Kleinbub, Paola Sessa. Interpretation of the data: Arianna Palmieri, Vincenzo Calvo, Johann R. Kleinbub, Paola Sessa. Drafting the manuscript: Arianna Palmieri, Vincenzo Calvo, Johann R. Kleinbub, Paola Sessa. Revising the manuscript for intellectual content: Arianna Palmieri, Vincenzo Calvo, Johann R. Kleinbub, Federica Meconi, Matteo Marangoni, Paolo Barilaro, Alice Broggio, Marco Sambin, Paola Sessa. Final approval of the version to be published: Arianna Palmieri, Vincenzo Calvo, Johann R. Kleinbub, Federica Meconi, Matteo Marangoni, Paolo Barilaro, Alice Broggio, Marco Sambin, Paola Sessa. Agreement to be accountable for all aspects of the work in ensuring that questions related to the accuracy or integrity of any part of the work are appropriately investigated and resolved: Arianna Palmieri, Vincenzo Calvo, Johann R. Kleinbub, Federica Meconi, Matteo Marangoni, Paolo Barilaro, Alice Broggio, Marco Sambin, Paola Sessa.

### Conflict of interest statement

Despite authors and Associate Editor being affiliated to the same institution as part of different departments, no conflict of interest exists and the review process was handled objectively. The authors declare that the research was conducted in the absence of any commercial or financial relationships that could be construed as a potential conflict of interest.
